# Ascorbic Acid 2-Glucoside Pretreatment Protects Cells from Ionizing Radiation, UVC, and Short Wavelength of UVB

**DOI:** 10.3390/genes11030238

**Published:** 2020-02-25

**Authors:** Junko Maeda, Allison J. Allum, Jacob T. Mussallem, Coral E. Froning, Alexis H. Haskins, Mark A. Buckner, Chris D. Miller, Takamitsu A. Kato

**Affiliations:** Department of Environmental & Radiological Health Sciences, Colorado State University, Fort Collins, CO 80523, USA; junkorv0507@yahoo.co.jp (J.M.); allison10allum@gmail.com (A.J.A.); jake.mussallem@gmail.com (J.T.M.); coral.froning@gmail.com (C.E.F.); 2013ahaskins@gmail.com (A.H.H.); mabuckner2@gmail.com (M.A.B.); cdmiller1668@gmail.com (C.D.M.)

**Keywords:** ascorbic acid 2-glucoside, narrowband UVB, broadband UVB, ionizing radiation, CHO, DNA damage

## Abstract

Ascorbic acid 2-glucoside (AA2G), glucosylated ascorbic acid (AA), has superior properties for bioavailability and stability compared to AA. Although AA2G has shown radioprotective properties similar to AA, effects for UV light, especially UVC and UVB, are not studied. AA2G was tested for cytotoxicity and protective effects against ionizing radiation, UVC, and broadband and narrowband UVB in Chinese hamster ovary (CHO) cells and compared to AA and dimethyl sulfoxide (DMSO). Pretreatment with DMSO, AA, and AA2G showed comparative protective effects in CHO wild type and radiosensitive xrs5 cells for cell death against ionizing radiation with reducing the number of radiation-induced DNA damages. Pretreatment with AA and AA2G protected CHO wild type and UV sensitive UV135 cells from UVC and broadband UV, but not from narrowband UVB. DMSO showed no protective effects against tested UV. The UV filtration effects of AA and AA2G were analyzed with a spectrometer and spectroradiometer. AA and AA2G blocked UVC and reduced short wavelengths of UVB, but had no effect on wavelengths above 300 nm. These results suggest that AA2G protects cells from radiation by acting as a radical scavenger to reduce initial DNA damage, as well as protecting cells from certain UVB wavelengths by filtration.

## 1. Introduction

Radioprotective agents are important tools for radiation protection. Since radiation induced damage is produced on the order of milliseconds after radiation exposure, the strategy for radioprotection is the administration of a radical scavenger before irradiation. Originally many agents were studied, and radioprotective properties of sulfhydryl residues in cysteine and cysteamine were identified [1,2]. Later, amifostine (WR-2721) was developed and approved for clinical use [3]. However, amifostine is the only clinically approved agent and still shows high toxicity. Therefore, investigation of novel radioprotectants is necessary for clinical and practical uses to protect patients from unwanted side effects and the general public from nuclear incidences.

Ascorbic acid (AA) is a well characterized antioxidant and is essential to the human body as Vitamin C [4]. It has been shown to have positive properties as a radioprotector. Pretreatment with AA protects cultured cells from ionizing radiation induced cell death, mutation, and other aspects as seen with other typical radioprotective agents. Interestingly, not only pretreatment, but post irradiation treatment, also showed some degree of radioprotective effects such as reduction of mutation frequency [5,6] and increase of cell survival [7] through scavenging of long-live radicals. Although these properties make AA a very interesting radioprotective agent, high doses of AA also show toxicity to cultured cells [8] and rapid secretion after intake by normal metabolism [9]. Ascorbic acid 2-glucoside (AA2G) is natural vitamin C stabilized with glucose, and was originally developed for use in cosmetic compounds. AA2G remains in the body longer than AA and showed evidence for less toxicity at high doses [10,11,12]. Glucosylation does not prevent AA’s radioprotective properties. Previous studies have shown in vitro and in vivo radioprotection effects of pre-treatment with AA2G in plasmid DNA, cultured cells, and mice after gamma-ray and fast neutron exposure [13,14,15,16,17]. In vivo, AA2G is hydrolyzed to express its potential AA activity [12].

In contrast to ionizing radiation protection, ultraviolet (UV) light protection occurs through several mechanisms. UV light produces pyrimidine dimers and other photoproducts along with oxidative stress. AA promotes collagen regeneration after UV induced depletion and reduces the minimal skin erythema dose after UV exposure [18]. As an antioxidant, AA quenches reactive oxygen species [19,20]. High levels of AA in the eye lens may suggest that AA contributes to protection of the eye through UV absorption [21]. AA2G is suggested to act in redox modulation as a protective mechanism against UVB [22,23]. Recently, ascorbic acid was found to reduce UV-induced apoptosis through reactivating silenced tumor suppressor genes [24]. Therefore, AA protects cells against UV light through multiple mechanisms.

This study expands those radioprotective properties of AA and AA2G, and investigates the protective effects of AA2G against UVC and UVB. Chinese hamster ovary (CHO) cell culture systems were used for this study. In order to identify the molecular and physical mechanisms of AA2G induced radiation and UV protection, analyses of gene deficient sensitive mutants and DNA damage were carried out.

## 2. Materials and Methods 

### 2.1. Cell Culture and Chemicals

Chinese hamster ovary (CHO) wild type CHO10B2, and Ku80 mutated radiosensitive xrs5 [25] and XPG mutated UV sensitive UV135 [26,27], AG1522 normal human fibroblast cells, and A549 lung cancer cells were kindly provided by Dr. Joel Bedford at Colorado State University (Fort Collins, CO, USA) and maintained at 37 °C in a 5% CO_2_ incubator in Eagle’s minimal essential medium with alpha modification supplemented with 10% heat-inactivated fetal bovine serum, penicillin and streptomycin. Ascorbic acid (Sigma, St Louis, MO, USA) and ascorbic acid 2-glucoside (AA2G, Toyo Sugar Refining Co. Ltd., Tokyo, Japan) were dissolved in phosphate buffer saline (PBS) for a 500 mM stock solution and used as 5 mM for a working solution.

### 2.2. Gamma-Ray and Ultraviolet Exposure

The cells were irradiated with gamma radiation at room temperature. For gamma ray irradiation, a J. L. Shepherd Model Mark I-68 nominal 6000 Ci Cs-137 sealed source (J.L. Shepherd, Carlsbad, CA, USA) was used as previously described [28]. Phillips germicidal UVC lamps (Phillips, Andover, MA, USA) were used for the UVC source, with a dose rate of 0.3 or 1 W/m^2^. Six Westinghouse Sunlamps (Westinghouse, Cranberry Township, PA, USA) were used for broadband UVB source, with a dose rate of 100 W/m^2^ [29], and one 40 W Phillips TL01 lamp was used for narrowband UVB source, with a dose rate of 1000 W/m^2^. Dosimetry was carried out using a UVP UVX dosimeter with UVC and UVB probes (UVP, Upland, CA, USA). Spectrums of each light were obtained using a ASEQ LR1 spectroradiometer (ASEQ Instruments, Vancouver, BC, Canada). The cells were cultured in P-30 Petri dishes and washed with PBS twice. Subsequently, 1 mL of PBS was added to prevent dryness during UV exposure without Petri dish lids unless specifically mentioned. Irradiation was carried out at room temperature.

### 2.3. In Vitro AA2G Properties

The total antioxidant capacity was obtained with Total Antioxidant activity kit (Sigma) as per the manufacturer’s instruction. Absorbance at 570 nm was obtained with Nanodrop spectrometer (Thermo Scientific, Wilmington, DE, USA). Trolox was used for standard. Antioxidant properties of AA2G were also analyzed with Ascorbic Acid Assay kit II (Sigma) as per the manufacturer’s instruction to compare with those of AA. Absorbance at 593 nm was obtained with a Nanodrop spectrometer after background subtraction.

### 2.4. Real-Time Cell Electronic Sensing (RT-CES)

Cellular cytotoxicity was analyzed with RT-CES method. Exponentially growing CHO10B2 (wild type) cells were plated with 20,000 cells per well in a gold-coated special 96-well E-plate (ACEA Biosciences, San Diego, CA, USA) for the impedance measurement. After cells were cultured in E-plates, cellular growth and viability were measured every 3 min for 72 h by the RT-CES Analyzer module. Dimethyl Sulfoxide (DMSO), AA and AA2G were added at the time of 43 h. Cell number was expressed as Cell Index (Arbitrary unit) from impedance measurement [30]. 

### 2.5. Cell Survival Analysis

Survival curves were obtained by a standard colony formation assay as previously described [31]. Immediately after exposure to radiation or UV light, cells were trypsinized. After trypsinization, the cells were plated in appropriate concentration onto 60 mm plastic tissue culture dishes and returned to the incubator for 7 days. The cultures were fixed with absolute ethanol and stained with 0.1% crystal violet. Lastly, colonies containing more than 50 cells were scored as survivors [17].

### 2.6. DNA Double Strand Break Measurements

DNA double strand break formation was analyzed with the constant field gel electrophoresis assay as previously described with CHO10B2 [32]. Gels were stained with Ethidium Bromide, and the intensity of DNA, which was retained in the plug and released into the lane, was measured by a Bio-rad ChemiDoc imager with Image Lab Software (Bio-Rad, Hercules, CA, USA). The fraction of DNA released was calculated from the amount of DNA in the plug and lane.

Indirect DNA double strand break formation was also analyzed with gamma-H2AX foci formation assay with relatively low dose [33]. In brief, contact inhibited G0/G1-phase AG1522 cells were treated with testing chemicals for 30 min before irradiation. Then, 30 min after irradiation, cells were fixed with 4% paraformaldehyde in PBS for 15 min followed by treatment with 0.2% Triton X-100 solution in PBS for 5 min. A mouse monoclonal antibody for anti-γ-H2AX (Millipore, Billerica, MA, USA) was diluted 1:500 and treated for one hour at 37 °C. Cells were then washed with PBS three times for 10 min each, and the secondary antibody (Alexa Fluor 488 conjugated goat anti-mouse, Invitrogen, Carlsbad, CA ,USA), diluted at a ratio of 1:500, was added for one hour. Slides were mounted in 4′,6-diamidino-2-phenylindole (DAPI) containing slow-fade (Invitrogen). Images were captured using a Zeiss Axioskop motorized z-stage fluorescent microscope (Zeiss, Jena, Germany) equipped with CoolSNAP HQ2 (Photometrics, Tucson, AZ, USA). Three independent experiments were performed. Manual counting was performed for foci analysis.

### 2.7. G2-premature Chromosome Condensation (PCC) Assay

A549 lung cancer cells were exposed to 1 Gy of gamma-rays. Just after irradiation, G2-PCC was induced by adding 50 nM calyculin [34]. Thirty minutes after incubation, cells were harvested and chromosome spreads were prepared as a standard protocol [35,36]. G2-PCC was analyzed with the frequency of chromatid type aberrations (chromatid types of gap, break and exchange) per cell. Fifty chromosome spreads were analyzed for three independent experiments.

### 2.8. Chromosomal Aberration Analysis

CHO cells were synchronized into G1 phase using the mitotic shake-off method [37]. Synchronized mitotic cells were subcultured in P30 dishes and incubated for two hours at 37 °C. Cells were treated with test compounds for 30 min and exposed to gamma-rays or UVC. Furthermore, 12 h after exposure, 0.2 μg/mL of colcemid (Invitrogen) was added to cells and allowed to incubate for an additional 6 h. Cells were harvested and then suspended in 4 mL of 75 mM KCl solution and placed in a 37 °C water bath for 20 min. A fixative solution of 3:1 methanol to acetic acid was added to the samples according to the standard protocol [35,36]. Giemsa stained metaphase chromosome images were taken using a Zeiss Axioplan microscope (Zeiss) equipped with Q-imaging Aqua CCD camera and Q-capture Pro software (QImaging, Surrey, BC, Canada). Fifty metaphase cells were scored for each treatment concentration. Data presented are the mean of chromosome type aberrations (dicentric chromosomes and centric ring chromosomes for gamma-rays and chromatid type exchanges for UVC) as a frequency per chromosome.

### 2.9. Filtration Analysis

UV block analysis was carried out with two methods. For spectrum filtration analysis, the testing chemical solution was filled in quartz optical cuvette with a 10 mm path, and the passed light spectrum was analyzed with the ASEQ LR1 spectroradiometer. For absolute filtration analysis, 15 mL conical tubes were cut and covered one side with an 1.5 μm thickness Myler membrane [38], which has minimal UV absorption. Tubes were filled with 1 mL of tested compounds and passed light strength was obtained with UVX radiometer with UVB or UVC probes.

### 2.10. Statistical Analysis

All experiments were carried out with three or more independent experiments. Data points were expressed as a mean with standard errors of the means. For statistical analysis, GraphPad Prism 5 software (San Diego, CA, USA) was used for a two-way ANOVA analysis and extra sum-of-squares *F*-test. Differences with *p* values of less than 0.05 were considered statistically significant.

## 3. Results

### 3.1. Antioxidant Effects

**Figure 1:**
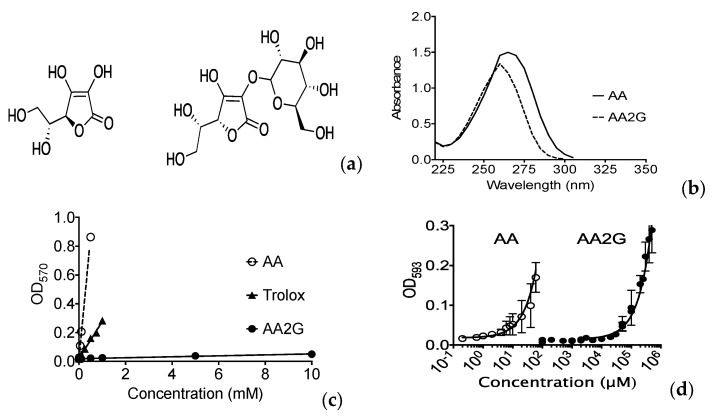
Chemical structures and basic characteristics of ascorbic acid and ascorbic acid 2-glucoside: (**a**) Chemical structures of ascorbic acid (AA) and ascorbic acid 2-glucoside (AA2G); (**b**) Absorption spectrum of 1 mM AA and AA2G solution; (**c**) In vitro total antioxidant capacity measured by reduction of Cu^2+^;(**d**) In vitro Ascorbic Acid specific antioxidant properties measured by reduction of Fe^3+^. Open circles indicate AA and closed circles indicate AA2G. Error bars indicate standard error of the means of three independent experiments.

Glucosylation modifies AA’s absorption spectrum (Figure 1a,b). The total antioxidant activities of AA and AA2G were analyzed with calorimetric analysis with reduction of Cu^2+^. Trolox was used for positive control, and glucose was used for negative control. AA showed much stronger total antioxidant capacity than AA2G in vitro (Figure 1c). Ascorbic acid specific ferric reducing assay was used to determine in vitro oxidative properties of AA2G. AA2G had a few thousand times lower antioxidant activity compared to AA (Figure 1d). Therefore, AA2G itself does not have antioxidant properties and required metabolic activation to get antioxidant properties as previously suggested [12].

### 3.2. Cytotoxicity Analysis with RT-CES

**Figure 2:**
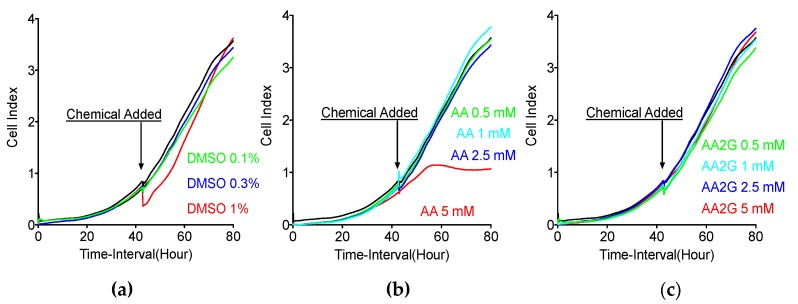
Dimethyl Sulfoxide (DMSO), AA and AA2G cytotoxicity measured by real-time cell electronic sensing (RT-CES) system. (**a**) DMSO (**b**) AA (**c**) AA2G, black solid line indicates control. Cell Index indicates relative cell number. Chemicals were added at 43 h after incubation. More than three experiments were carried out. Error bars were omitted.

Cytotoxic effects from continuous exposure to chemicals were measured with an automatic impedance change system. It was noted that 0.1% and 0.3% DMSO treatment did not change Cell Index. Although 1% DMSO showed a rapid decrease in Cell Index, Cell Index was recovered later (Figure 2a). A sudden decrease of Cell Index was due to cellular morphological and attachment changes with 1% DMSO. Moreover, 5 mM AA treatment showed a marked delay in cell growth immediately after treatment began, as observed in a slower rate of increasing Cell Index and a decrease of Cell Index at 12 h after treatment started. Such cytotoxic responses were not observed with 0.5, 1 and 2.5 mM AA treatment (Figure 2b). Cells were growing without any noticeable change of Cell Index with 0.5, 1, 2.5 and 5 mM AA2G treatment starting at 43 h after cell culture (Figure 2c). This result revealed that cells may tolerate short term exposure. Indeed, clonogenic ability measured by colony formation assay was not affected by short term chemical exposure to CHO10B cells. Plating efficiency values of control, 1% DMSO, 5 mM AA and 5 mM AA2G, were 73 ± 9.8, 70 ± 7.6, 70 ± 8.8, and 73 ± 9.2 (mean % ± standard deviation), respectively. Based on these results, this study used 5 mM of AA and AA2G, and 1% DMSO as a 30 min short term pre-exposure before irradiation.

### 3.3. Cell Survival after Gamma-Rays

**Figure 3:**
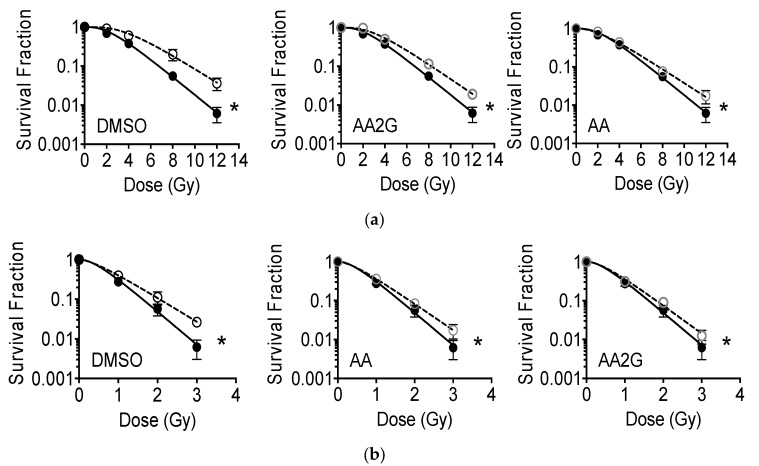
Cell survival curves, cells treated with DMSO, AA, AA2G before gamma-ray exposure. (**a**) CHO10B2 cells; (**b**) xrs5 cells. Closed circle symbols with solid lines indicate non-drug treated control. Open circle symbols with dashed lines indicate data with chemical pre-treatment. Error bars indicate standard error of the means of at least three independent experiments. * indicates statistically significant differences between control and chemical treatment (*F*-test, *p* < 0.05).

Exponentially growing CHO10B2 (wild type) and xrs5 (Ku80 mutant) were exposed to gamma-rays with pre-treatment of 1% (*v*/*v*) DMSO, 5 mM AA, or 5 mM AA2G for 30 min, and were tested for radioprotective effects using a colony formation assay. The dose response for cell survival curve is shown in Figure 3. All three agents showed radioprotective effects in CHO wild type survival compared to non-treated control (Figure 3a). Radiosensitive xrs5 cells were also protected by pre-treatment with DMSO, AA, and AA2G (Figure 3b). Although statistically significant increases of survival fraction were observed at DMSO pretreated 8, 12 Gy for CHO and 3 Gy for xrs5 (one-way ANOVA). An extra sum-of-squares F test showed statistically significant differences between control and chemicals (*p* < 0.05).

The D_10_ values, a dose to kill 90% cell population, are summarized in Table 1. The degree of protective effects, measured as the protection ratio, was calculated by D_10_ (pre-treated with chemicals) over D_10_ (non-treated control). Among the three chemicals, the protection ratio was the highest with DMSO pretreatment (1.42), but the ratio for each chemical did not change between CHO wild type and xrs5. It suggests that the three chemicals did not protect cells by enhancing DNA repair capability, but rather through other pathways such as reducing initial DNA damage.

### 3.4. DNA Damage Induction after Gamma-Ray Exposure

**Figure 4:**
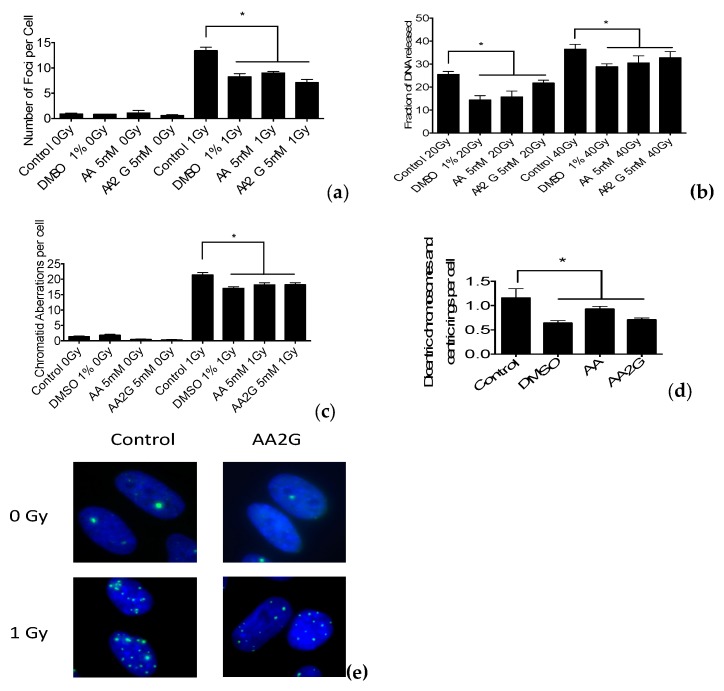
Effects of DMSO, AA, and AA2G for the radiation induced damages: (**a**) Changes in DNA double strand breaks measured by gamma-H2AX foci immunostaining assay at 30 min after irradiation with AG1522 cells; (**b**) Changes of initial DNA double strand breaks measured by constant field gel electrophoresis as a fraction of DNA released to gel with CHO10B2 cells; (**c**) Chromatin DNA damage measured by G2-premature chromosome condensation at 30 min after irradiation with A549 cells; (**d**) Chromosomal aberration analysis. Cells in G1 phase were irradiated with 4Gy and first post irradiation metaphase chromosomes were analyzed with CHO10B2. Error bars indicate standard error of the means of three independent experiments. * means statistically significant differences (*p* < 0.05). (**e**) representative images of gamma-H2AX foci (green) and nuclear stain with DAPI (blue).

DNA double strand breaks were analyzed using two methods. The γH2AX foci formation assay was used with a relatively low dose (1 Gy) for indirect immunochemical analysis of DNA double strand breaks. On the other hand, the constant field gel electrophoresis assay was used with a relatively high dose (40 Gy) for direct measurement of double strand breaks. Both assays suggested a reduction in the initial number of DNA double strand breaks for the three chemicals. γH2AX foci reached a maximum number of approximately 14 foci per cell at 30 min after irradiation, and an approximately 25% reduction in the number of foci was observed compared to the control (Figure 4a). Constant field gel electrophoresis measures DNA double strand breaks by the fraction of DNA released (FDR). The FDR of samples pre-treatment with radioprotective chemicals showed approximately a 10% to 40 % reduction in FDR compared to non-pretreated samples (Figure 4b). A549 cells were pre-treated with chemicals and exposed to gamma-rays. G2-PCC by calyculin A was induced just after irradiation. G2-chromatid aberrations were scored as number of chromatid gaps and breaks per cell. Figure 4c shows pre-treatment with chemicals reduced the number of chromatid breaks. Therefore, the three assays support the proposal that tested chemicals protect cells from ionizing radiation by reducing DNA damage formation.

### 3.5. Chromosomal Aberrations after Gamma-Ray Exposure

G1 synchronized CHO10B2 cells were exposed to gamma-rays with and without radioprotective chemicals. First, post-irradiation mitotic metaphase cells were analyzed for chromosomal aberrations. The number of dicentric and centric ring chromosomes per cell were analyzed with pre-treated samples, showing reduced numbers of chromosomal aberrations (Figure 4d). It suggests that initial DNA damage is reduced by radioprotective agents which suppress the formation of lethal chromosomal aberrations and protect cells from ionizing radiation.

### 3.6. Filtration of UV Light 

**Figure 5:**
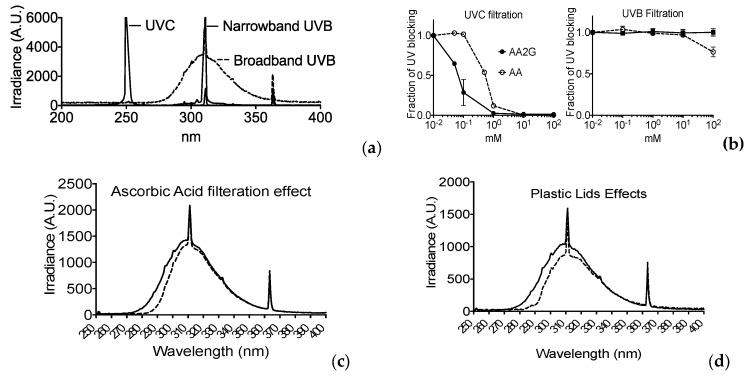
Spectrum of each UV light source and AA and AA2G induced UV filtration analysis: (**a**) Energy spectrum of each UV light source; (**b**) UV reduction analysis with radiometer. Error bars indicate standard error of the mean; (**c**) 1 mM AA induced filtration effects for broadband UVB by spectrum analysis with spectroradiometer. Solid lines indicate spectrum without AA. Dashed lines indicate AA filtered spectrum; (**d**) Plastic lids induced filtration effect for broadband UVB by spectrum analysis with spectroradiometer. A solid line indicates the spectrum without a plastic lid. A dashed line indicates the spectrum with the plastic lid filter.

The light spectrum of each UV light was obtained by spectroradiometer and is illustrated in Figure 5a. The absorption spectrums of AA and AA2G measured with the spectrometer are shown in Figure 1b. AA showed an absorption spectrum up to 300 nm, and AA2G showed one until 280 nm. AA and AA2G induced UV light filtrations were analyzed using two methods. Firstly, the energy filtration of each was analyzed using the spectrometer with UVB, and UVC specific probes and quartz optical cuvette with a 10 mm path to measure total energy for the specific UV spectrum of light (Figure 5b). UVC light was completely blocked by 1 mM of AA and AA2G, with AA2G showing better UVC filtration capacity than AA. However, 1% DMSO did not show any filtration effect for UVC. Filtration of UVB light was not observed with AA, AA2G, or DMSO (Figure 5b). However, this result does not match the UVB protective effects of AA and AA2G (Figure 3a,b). Secondly, a detailed filtration analysis of the spectrum was carried out with a spectroradiometer for broadband UVB. AA showed a reduction in the short wavelength component of broadband UVB below 300 nm (Figure 5c). The energy fraction is very minimal compared to total broadband UVB energy. Therefore, a simple spectrometer could not detect the significant differences with a UVB specific probe. The UVB spectrum was also selectively blocked in the shorter wavelengths when UV light passed through plastic lids (Figure 5d).

### 3.7. Cell Survival after UVC

**Figure 6:**
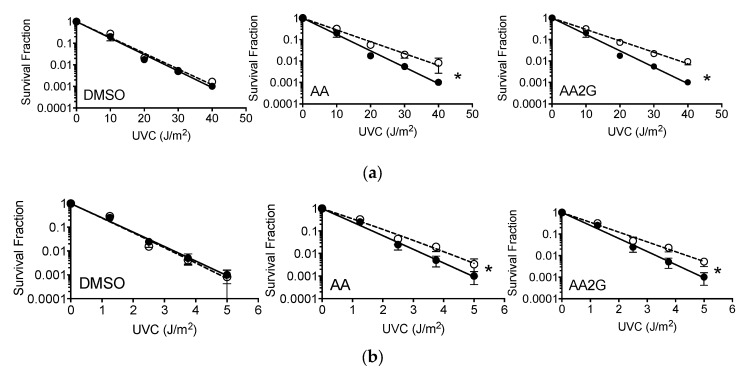
Cell survival curves, cells treated with DMSO, AA, AA2G before UV light exposure: (**a**) UVC exposed CHO10B2; (**b**) UVC exposed UV135. Closed circle symbols with solid lines indicate non-drug treated control. Open circle symbols with dashed lines indicate data with chemical pre-treatment. Error bars indicate standard error of the mean. * means statistically significant differences (*F*-test, *p* < 0.05).

Exponentially growing CHO10B2 (wild type) and UV135 (XPG mutant) were exposed to UVC after pre-treatment with 1% (*v*/*v*) DMSO, 5 mM AA, or 5 mM AA2G for 30 min and were examined for cytotoxic effects by colony formation assay. The dose response for cell survival curve is shown in Figure 6a,b. AA and AA2G showed UV-protective effects in CHO wild type survival compared to non-treated control (protective ratio 1.38 and 1.44 respectively, Table 2). DMSO did not protect cells from UVC. UV-sensitive UV135 cells were also protected by pre-treatment with AA, or AA2G but not DMSO. The protection ratio did not change between CHO wild type and UV135 (Table 2). It may suggest that the three chemicals did not protect cells by enhancing DNA repair capability but by affecting initial DNA damage.

### 3.8. Chromosomal Aberrations after UVC Exposure

**Figure 7:**
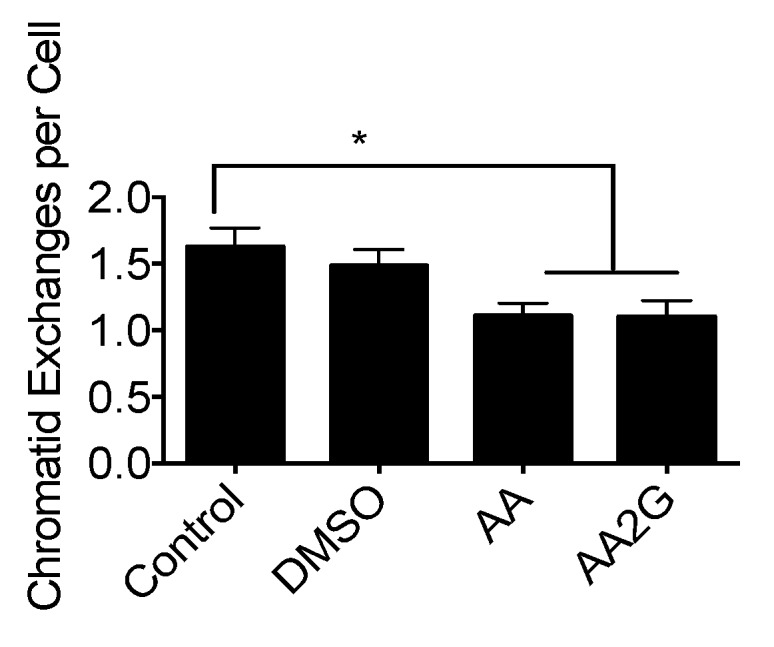
Effects of DMSO, AA, and AA2G for the UVC induced damages: Chromosomal aberration analysis, G1 exposed first post irradiation metaphase with 10 J/m^2^ UVC. Error bars indicate standard error of the mean. * indicates statistically significant differences (*p* < 0.05).

G1 synchronized CHO10B2 cells were exposed to UVC with and without radioprotective chemicals. First post-irradiation mitotic metaphase cells were analyzed for chromosomal aberrations. The numbers of chromatid type exchanges per cell were analyzed. Pre-treatment with AA or AA2G showed a reduction in the number of chromosomal aberrations (Figure 7). This suggests that initial DNA damage reduced by UV-protective agents suppresses the formation of lethal chromosomal aberrations and protects cells from UVC.

### 3.9. Cell Survival after Broadband and Narrowband UVB

**Figure 8:**
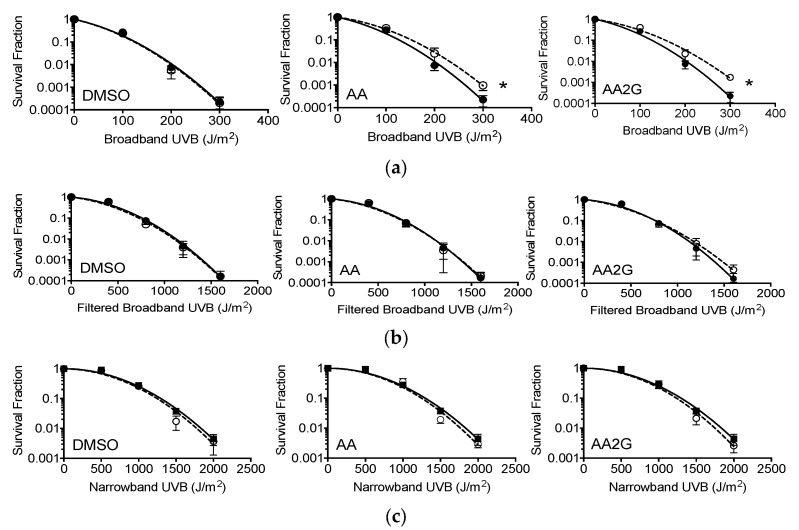
Cell survival curves, cells treated with DMSO, AA, AA2G before UVB light exposure: (**a**) Broadband UVB exposed CHO10B2 without plastic lids; (**b**) Broadband UVB exposed CHO10B2 with plastic lids; (**c**) Narrowband UVB exposed CHO10B2. Closed circle symbols with solid lines indicate non-drug treated control. Open circle symbols with dashed lines indicate data with chemical pre-treatment. Error bars indicate standard error of the mean. * means statistically significant differences (*F*-test, *p* < 0.05).

Exponentially growing CHO10B2 (wild type) and UV135 (XPG mutant) were exposed to broadband (with and without plastic lids) or narrowband UVB with pre-treatment with 1% (*v*/*v*) DMSO, 5 mM AA, or 5 mM AA2G for 30 min and were tested for cytotoxic effects by colony formation assay. The dose response for cell survival curve is shown in Figure 8a–c. D_10_ values and protection ratio are summarized in Table 3. The cytotoxic effects were the strongest in broadband UVB without lids, followed by broadband UVB with lids. Narrowband UVB was the least toxic among the three UVB lights tested in this study. AA and AA2G showed protective effects only for broadband UVB without lids in CHO wild type survival compared to non-treated control. On the other hand, neither AA nor AA2G protected cells from broadband UVB with lids and narrowband UVB exposure. DMSO did not protect cells from broadband or narrowband UVB.

## 4. Discussion

DMSO, AA and AA2G have been studied for their radioprotective properties [15,39]. They share molecular mechanisms for the protection against ionizing radiation. Our study confirmed that antioxidant AA and AA2G have the ability to act as a radical scavenger (Figure 1) and to protect cells from ionizing radiation through reduction of initial DNA damage in different types of mammalian cells (Figure 3 and Figure 4). The study of radioprotective agents has contributed not only to the development of radioprotectors for practical use, but also to understanding the mechanism of action for ionizing radiation on cells.

Unlike in the case of ionizing radiation, protection against ultraviolet light has controversial mechanisms. This is because the mechanism of cell death after being exposed to ultraviolet light can be governed by several pathways and is still disputed. It has been clearly reported that UV produces different types of DNA damage than ionizing radiation [40]. It is known that UV light leads to base modifications from oxidative stress and produces photoproducts such as thymine dimers via light excitation. Therefore, many researchers agree with the concept that UV light kills cells though oxidative stress, and because of this mechanism, antioxidants can protect cells by neutralizing oxidative damage produced by UV light.

This study showed that DMSO, one of the longest studied and best radioprotective agents and hydroxyl radical scavengers, had no protective effects against UVC and UVB exposure (Figure 6 and Figure 8). Ionizing radiation produces hydroxyl radicals and DMSO is a hydroxyl radical scavenger [41]. However, hydroxyl radicals are not associated with UV induced cell death. On the other hand, AA and AA2G showed positive protective effects against specific UV wavelengths (less than 300 nm wavelength), but not against wavelengths of more than 300 nm. Shorter wavelength UV light is more toxic because of quantitative differences in DNA damage but not qualitative DNA damage [42,43]. Our results suggest that wavelength specific protection by AA and AA2G can be through indirect biochemical antioxidant reactions of AA and AA2G, but also through direct interaction between UV light and AA and AA2G.

Our absolute light energy filtration experiment in vitro showed almost complete blockage of UVC light with AA and AA2G in relevant concentrations (0.1 mM) and matched with cellular protective effects (Figure 5 and Figure 6). Although we observed minimum filtration effects with absolute energy of broadband UVB, AA and AA2G protected cells from broadband UVB. These conflicting results were solved with further spectrum analysis of UVB light with AA and AA2G. Although glucosylation of AA shifts the absorption spectrum to shorter wavelength (Figure 1b) as previously reported [12], AA2G most likely presents in cells as AA. Therefore, in this filtration study, only AA results were reported (Figure 5c). As seen in the absorption spectrum, AA selectively blocked shorter wavelengths of broadband UVB. At a high concentration, AA can preferentially filter wavelengths shorter than 300 nm. Although broadband UVB contains extremely small amounts of short wavelengths, shorter than 300 nm (less than 5% of total irradiance), these shorter wavelengths of UVB have much stronger cytotoxic effects through the production of UV damage. Adding plastic lids during exposure mimicked the filtration of short wavelength of UVB (Figure 5d and Figure 8b) as previously reported [29]. Due to the fact that lids absorb the majority of short wavelength UVB, when lids were used during irradiation, no AA or AA2G induced UVB protection was observed (Figure 8). Since this is a small portion of UVB, absolute energy analysis could not detect large differences (Figure 5b).

## 5. Conclusions

In summary, this study showed that AA2G has the ability to be a radioprotector and has positive protective effects for UVC and the short wavelength region of UVB. The radioprotective mechanism of AA2G is through radical scavenging, but UV protection was based on filtration of specific wavelengths of UV. This study did not focus on post irradiation protection with AA2G in mutation and apoptosis reduction. AA2G might be a good radioprotector and UVC/UVB protector. Further studies in post-irradiation analysis and in vivo research should prove whether AA2G is an effective radioprotectant in humans.

## Figures and Tables

**Table 1:** D_10_ values and protection ratio of gamma-rays.

Cells	D_10_ of Gamma-Ray (Gy)/Protection Ratio			
	Control	DMSO	AA	AA2G
CHO	6.7	9.5/1.4	7.6/1.1	8.2/1.2
xrs5	1.6	2.1/1.3	1.9/1.2	1.8/1.1

**Table 2:** D_10_ values and protection ratio of UVC.

Cells	D_10_ of UVC (J/m^2^)/Protection Ratio			
	Control	DMSO	AA	AA2G
CHO	13.1	13.7/1.1	18.1/1.4	18.8/1.4
UV135	1.66	1.63/1.0	2.16/1.3	2.20/1.3

**Table 3:** D_10_ values and protection ratio of UVB.

Conditions	D_10_ of UVB (J/m^2^)/Protection Ratio			
	Control	DMSO	AA	AA2G
Broadband UVB	126	124/0.98	151/1.20	156/1.23
Broadband UVB + filter	758	722/0.95	741/0.98	763/1.01
Narrowband UVB	1297	1248/0.96	1248/0.96	1236/0.95
